# MICROBIOME MODULATORS IN THE PREVENTION AND MITIGATION OF CANCER TREATMENT-INDUCED ACCELERATED AGING BIOLOGY

**DOI:** 10.1093/geroni/igad104.2993

**Published:** 2023-12-21

**Authors:** Brandi Miller, Sidharth Mishra, Diptaraj Chaudhari, Hariom Yadav, Shalini Jain

**Affiliations:** University of South Florida, Tampa, Florida, United States; University of South Florida, Tampa, Florida, United States; University of South Florida, Tampa, Florida, United States; University of South Florida, Tampa, Florida, United States; University of South Florida, Tampa, Florida, United States

## Abstract

Advancements in oncology research have led to increased survivorship in patients with cancer. However, cancer treatments like chemotherapy cause debilitating side effects including multi-organ dysfunction and increased risk for chronic diseases, which mimic accelerated aging. Abnormalities in the gut microbiome composition (dysbiosis) increase inflammation, resulting in accelerated cognitive impairment with aging and a poor quality of life (QOL), which are all exacerbated by cancer treatments. We have screened, evaluated, and optimized our previously isolated human-origin live bacteria (probiotics) as well as pasteurized probiotics (postbiotics) for their anti-aging potential using Caenorhabditis (C.) elegans and mouse models. Probiotics improved the healthspan in C. elegans when given live and when fermented. Interestingly, PFM and LpD3.5 significantly protected from body weight loss, microbiome dysbiosis, and cognitive impairment in mice receiving 5-fluorouracil chemotherapy and protected from intestinal damage. We are planning a clinical trial where we will (1) cross-sectionally analyze the gut microbiome, leaky gut, and inflammation in cancer survivors with and without brain distress and (2) determine the safety, tolerance, and efficacy of PFM and LpD3.5 on microbiome diversity, inflammation, and cognitive health in cancer survivors with brain distress, using a 60-day open-label, randomized comparison study. These studies will improve our understanding of how cancer treatment-induced changes in the gut microbiome, inflammation, and gut permeability contribute to accelerated aging. Furthermore, they will establish the utilization of probiotics and postbiotics to improve QOL and mitigate cancer treatment-induced accelerated aging, which constitutes a debilitating public health concern among the increasing population of cancer survivors.

